# Improving the Collection of National Health Data: The Case for the Middle Eastern and North African Checkbox in the United States

**DOI:** 10.21203/rs.3.rs-2790994/v2

**Published:** 2023-04-20

**Authors:** Tiffany B Kindratt

**Affiliations:** University of Texas at Arlingtion

**Keywords:** Middle Eastern and North African, Arab American, US Census

## Abstract

**Objectives:**

To describe public comments posted in relation to the Office of Management and Budget (OMB) Statistical Policy Directive 15 proposals regarding the addition of a separate Middle Eastern and North African (MENA) checkbox on the US Census and other required federal forms.

**Methods:**

A public comment period outlining changes to the collection of race and ethnicity data on the US Census and other federal forms opened in January 2023. Public comments posted in February and March 2023 were reviewed to determine whether MENA was mentioned, whether comments supported a MENA checkbox, and whether comments mentioned support for health-related reasons.

**Results:**

There were 3,062 comments reviewed. Most (71.49%) mentioned adding a MENA checkbox. Of those, 98.86% supported adding a MENA checkbox. Among those, 31.98% mentioned adding a MENA checkbox for health-related reasons.

**Conclusions:**

Overall, the comments reviewed demonstrated strong support for the addition of a MENA checkbox on federal forms. These findings are encouraging yet further review is needed to contribute to the OMB’s final decision on whether to add the checkbox and uncover the health of this underrepresented population.

## Introduction

The ability to collect national health data on Middle Eastern and North African (MENA) Americans is in our nation’s reach. On June 15, 2022, the Chief Statistician of the United States (US) announced that the Office of Management and Budget (OMB) would conduct a formal review of the current minimum standards for collecting race and ethnicity data on the US Census and other federal forms.^1^ The current standards, outlined in Statistical Policy Directive No. 15 (SPD 15), have not been updated since 1997 and used to allocate billions of dollars of federal funds to communities for arts, education, health research, and other programs.^2^ The review involves the development of workgroup, public listening sessions, and an open comment period for the public to provide feedback.^1^ Several themes emerged from initial listening sessions during Fall 2022, including the need for data on MENA populations.^3^ The target date for completing the revision is Summer 2024.^1,4^

SPD 15 requires six minimum reporting categories, including: American Indian/Alaskan Native; Asian; Black/African American; Hispanic/Latino; Native Hawaiian/Other Pacific Islander; and White.^5^ The White category includes a heterogeneous group of individuals with origins from Europe, the Middle East, and North Africa.^5^ Classifying MENA individuals as White is concerning given that research demonstrates their health behaviors,^6–8^ disability/limitations,^9,10^ health conditions,^11,12^ mortality^13,14^ and lived experiences^15^ differ from Whites. Many MENA individuals do not perceive themselves as White nor do others perceive them as White.^15^ The initial OMB proposal was published in January 2023, with request for public comment for 90 days. It outlines six specific proposals for consideration, including the addition of new MENA checkbox as part of a combined race/ethnicity question. OMB requests public comment on the acceptability of the term MENA, the adequacy of the examples provided as subcategories (Lebanese, Iranian, Egyptian, Syrian, Moroccan, Israeli), and whether the definition will accurately reflect the well-being of the population.

In this paper, we provide an initial study of the public comments posted to determine the proportion of comments that mention including a MENA checkbox, support including a MENA checkbox, and mention support for health-related reasons.

## Methods

Comments posted on the Federal Reserve website^3^ from February 1-March 1, 2023 were reviewed to determine whether MENA was mentioned. Search terms included “MENA,” “Middle East,” “North Africa,” “Arab,” and countries or ethnic groups in the region identified by the US Census Bureau (e.g., Iran, Israel, Lebanon, Palestine, Chaldean). Comments that mentioned MENA were reviewed to determine whether they indicated support for the checkbox. Comments that supported a MENA checkbox were evaluated to determine whether health-related topics were mentioned. Basic search terms included “health,” “healthcare,” “health care,” “medicine,” “medical,” and “research.” Frequencies and percentages were used to describe the findings. The institutional review board deemed this project using publicly available data not subject for review.

## Results

Initial findings are presented in Table 1. There were 3,062 comments reviewed that were posted on the Federal Registrar’s website from February 1-March 1, 2023. Among the comments reviewed, most (71.49%) mentioned the addition of a MENA checkbox. Of those, 98.86% supported adding the MENA checkbox and 31.98% acknowledged the need for a MENA checkbox for health-related reasons.

A full qualitative analysis of comments is beyond the scope of this assessment. In general, common themes related to adding a MENA checkbox for health-related reasons specifically mentioned the following topics: cancer screenings; health behaviors; influenza; COVID-19; functional disability; mental health; and maternal mortality.

## Discussion

The purpose of this paper was to describe the initial comments submitted to the OMB related to adding a separate MENA checkbox for race/ethnicity. Overall, findings showed strong support for adding a MENA checkbox. Implications for health research are provided below.

There are only two national surveys that allow for MENA health outcomes to be determined, the American Community Survey (ACS) and National Health Interview Survey (NHIS). ACS collects monthly samples to produce annual national estimates of demographic and socioeconomic factors from a random sample of households.^16^ MENA individuals have been identified using questions on race, ethnicity, place of birth, and ancestry in previous research.^17,18^ However, ACS only collects information on health insurance, health care access, and disability.^16^ From 2000–2018, NHIS allowed for the assessment of several health topics among MENA individuals. NHIS collects a wide range of sociodemographic, health care access, health behaviors, and health outcomes from a representative US sample.^19^ Foreign-born MENA individuals have been identified using questions on race, ethnicity, and country of birth in previous research.^7,8,11^ However, the NHIS was redesigned in 2019 and questions on country of birth were removed.^19^ Other national health surveys do not collect data on MENA individuals.

Among the comments submitted in support of a MENA category, 31.98% mentioned support for the checkbox for the collection of health-related topics. Health-related topics mentioned most were cancer screening, health behaviors, influenza, COVID-19, functional disability, mental health, and maternal mortality. On a national level, functional disability is the only one of these topics that can currently be measured for MENA individuals using ACS data. Until 2019, other health topics could be measured among foreign-born MENA individuals using NHIS data, but with the removal of the question on country of birth, current and future trends cannot be examined. In 2021, the NHIS included questions on COVID-19.^19^ Without the accompanying country of birth question, national estimates for MENA individuals will not be able to be measured.

It is important to acknowledge that including a MENA checkbox may not only provide health data for MENA populations, but it may also allow for a truer assessment of health disparities between other populations and White individuals.^20^ By removing MENA from the White category, research studies may find a lower prevalence of specific health burdens among White individuals and create a larger disparity between other minority groups and White individuals. Future studies on MENA health should consider using methods to remove MENA individuals from the White category and make comparisons with all other racial/ethnic groups required for federal reporting to further the science on health disparities.^20^

This is one of the first attempts to compile comments related to adding a MENA checkbox during the OMB’s 2023 request for public comments on SPD 15. The analysis was purely descriptive and limited to comments posted during the first month of the comment period. As of this writing, it represents 23.24% of the public comments currently posted (n=13,172). This research team will continue to review and compile comments and expect that similar trends will appear when all comments are analyzed. We selected to compile the first month of comments for review to contribute to the timely scientific dialogue and advocacy efforts. Future analyses of the comments should fully address other concerns mentioned by individuals who supported a MENA checkbox, such as its support for small businesses, education, linguistic services and voting rights.

## Figures and Tables

**Table 1.** Description of initial OMB public comments posted, n=3,062.

	Frequency *n=3,062*	%
Comments acknowledges adding a MENA checkbox	2,189	71.49
Supports MENA checkbox	2,164	98.86
Supports MENA checkbox for health data	692	31.98

Abbreviation: MENA=Middle Eastern and North African

## PUBLIC HEALTH IMPLICATIONS

Adding a MENA checkbox will allow for the first comprehensive nationally representative baseline assessment of the health needs of MENA adults and children. Furthermore, removing MENA from the White race group is needed to improve scientific rigor for health equity research between other racial/ethnic groups and Whites. The results of this study indicated strong support for the MENA checkbox. While the findings are encouraging, it remains unknown whether the OMB will make changes to uncover the health needs of this vulnerable underrepresented population.
